# Volume Analysis to Predict the Long-Term Evolution of Residual Aortic Dissection after Type A Repair

**DOI:** 10.3390/jcdd9100349

**Published:** 2022-10-12

**Authors:** Marine Gaudry, Carine Guivier-Curien, Arnaud Blanchard, Alizée Porto, Laurence Bal, Virgile Omnes, Mariangela De Masi, Charlotte Lu, Alexis Jacquier, Philippe Piquet, Valerie Deplano

**Affiliations:** 1 Timone Aortic Center, Department of Vascular Surgery, APHM, Timone Hospital, 13005 Marseille, France; 2 CNRS, Centrale Marseille, IRPHE, Aix Marseille University, 13013 Marseille, France; 3 Department of Cardiac Surgery, APHM, Timone Hospital, 13005 Marseille, France; 4 Department of Radiology, APHM, Timone Hospital, 13005 Marseille, France

**Keywords:** type A aortic dissection, residual aortic dissection, volume analysis, predictive factors, long term results

## Abstract

Background: The aim of this study was to evaluate the aortic diameter and volume during the first year after a type A repair to predict the long-term prognosis of a residual aortic dissection (RAD). Methods: All patients treated in our center for an acute type A dissection with a RAD and follow-up > 3 years were included. We defined two groups: group 1 with dissection-related events (defined as an aneurysmal evolution, distal reintervention, or aortic-related death) and group 2 without dissection-related events. The aortic diameters and volume analysis were evaluated on three postoperative CT scans: pre-discharge (T1), 3–6 months (T2) and 1 year (T3). Results: Between 2009 and 2016, 54 patients were included. Following a mean follow-up of 75.4 months (SD 31.5), the rate of dissection-related events was 62.9% (34/54). The total aortic diameters of the descending thoracic aorta were greater in group 1 at T1, T2 and T3, with greater diameters in the FL (*p* < 0.01). The aortic diameter evolution at 3 months was not predictive of long-term dissection-related events. The total thoracic aortic volume was significantly greater in group 1 at T1 (*p* < 0.01), T2 (*p* < 0.01), and T3 (*p* < 0.01). At 3 months, the increase in the FL volume was significantly greater in group 1 (*p* < 0.01) and was predictive for long-term dissection-related events. Conclusion: This study shows that an initial CT scan volume analysis coupled with another at 3 months is predictive for the long-term evolution in a RAD. Based on this finding, more aggressive treatment could be given at an earlier stage.

## 1. Introduction

The long-term prognosis of a type A aortic dissection is directly related to the residual aortic dissection (RAD) evolution [1,2]. Indeed, a patent false lumen (FL) in the descending aorta is the most common situation encountered after the replacement of the ascending aorta for a type A aortic dissection (TAAD) and is a well-known risk factor for aortic growth, reinterventions, and mortality [3,4,5,6,7]. The identified poor prognostic factors associated with a RAD are a matter of concern because patients with an increased risk of disease progression might be offered earlier endovascular treatment to increase the mid- and long-term survival.

Currently, the most relevant anatomical criteria found in the literature were the initial aortic diameter and the presence of a new entry tear on the distal anastomosis or the absence of a resection of the primary entry tear [3,4,5,6,8,9,10,11,12,13]. However, different authors have stated that using the diameter measurements is not sensitive and adequate to evaluate the behavior of an aortic dissection due to the long extent of the aorta and its pathology [14] and, although time consuming, that aortic volume measurement was the most relevant and consistent method to reliably assess an aortic remodeling during a follow-up [15,16,17,18,19]. The volumetric measurement has been largely studied in abdominal aneurysm repair [20], but there are few studies on volume analysis with the thoracic aorta [15] and in an aortic dissection or other thoracic aortic diseases, the role of volume measurement remains unknown.

The aim of this study was to investigate the aortic diameter and volume during the first year after a type A repair to predict the long-term prognosis of a RAD.

## 2. Patients and Methods

All patients included in this study were informed of the use of their data for clinical research, and the institutional review board approved the project (approval number 2019–48).

### 2.1. Inclusion and Exclusion Criteria

Between January 2010 and December 2016, all patients treated in our center for a TAAD with a RAD, a radiological and clinical follow-up > 3 years and three early CT postoperative scans suitable for radiological analysis (pre-discharge (T1), 3–6 months (T2) and 1 year (T3) CT scan) were included in this study.

### 2.2. Radiological Analysis

#### 2.2.1. Diameter Measurement

**Figure 1:**
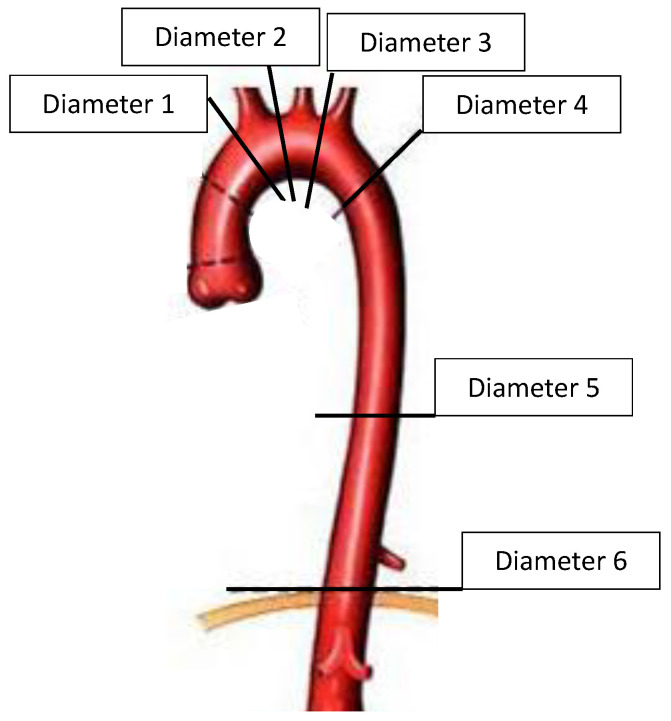
Levels of the diameter measurements.

Diameter measurements were performed using Endosize software (Therenva version 3.1.25, Rennes, France) in the perpendicular axis of the centerline (CL) at six levels for the true lumen (TL), false lumen (FL), and the total aortic diameter of the thoracic aorta (Figure 1).

To assess the intra- and interobserver variability of the aortic diameter measurements, a random sample of 50 CT scans was analyzed a second time by the same observer and by a second radiologist.

#### 2.2.2. Entry Tear

The number of entry tears on each CT-scan was reported, and its diameter measured.

#### 2.2.3. Volume Calculation

**Figure 2:**
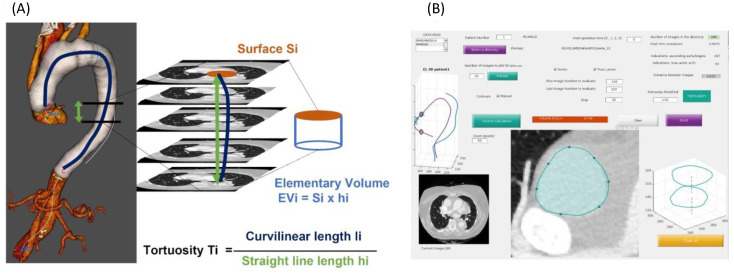
Method for the volume measurement with the TAV method. (**A**): Diagram showing the volume calculation method with MATLAB. (**B)**: The interface used for the volume measurement.

Custom MATLAB application (Figure 2).

We developed a custom application through the MATLAB software (named TAV (thoracic aorta volume)) to evaluate the volumes of the thoracic aorta.

The method is based on the addition of the elementary volume (EV). It is assumed that the area of a surface (S_i_) that is identified on an axial CT scan image does not vary over a certain height h_i_. This hypothesis is valid if the EVi remains straight, i.e., its tortuosity (Ti) remains low. Ti is the ratio between the EV curvilinear length (li) and its straight-line length (hi). The height is optimal when the tortuosity is close to 1. A maximum tortuosity threshold is therefore chosen to validate the hypothesis of a constant area over h_i._ The elementary volume V_i_ is then equal to Si x hi. The total volume is the sum of all of the elementary volumes over the segment of interest.

The main advantage of this method is that it allows a measurement of the volumes of the TL and FL in 30 min (compared to 1 h with the standard method), which is less expensive (compared to the available software) and can be performed on any computer.

#### 2.2.4. Volume Calculation Validation: Semiautomated Method Coupled with the Standard Method

To validate the custom application of the TAV, the results were compared with those results obtained when using a reference method made by another observer [21,22,23].

The volume analysis was performed with semiautomated segmentation, which determines the boundaries around the voxels with a similar intensity (TL and contrast aortic lumen). For the FL, a manual selection was carried out at each slice.

To assess the intra- and interobserver variability of the aortic volume measurements, a random sample of 50 CT scans was analyzed a second time by the same observer and by a second radiologist.

## 3. Endpoints

We defined two groups: group 1 with dissection-related events defined as (i) aneurysmal evolution during the 3 years of follow-up (aortic growth > 10 mm), (ii) distal reintervention (aortic diameter greater than 55 mm, rapid aortic growth (10 mm/y), malperfusion syndrome or aortic rupture) or aortic-related death; and group 2 without dissection-related events during the 3-year follow-up.

We analyzed the aortic diameters and the volume at T1, T2 and T3.

We analyzed the aortic diameter and the volume evolution: the change between the postoperative and the 3-month CT scans and between the postoperative CT scans and the 1-year CT scans.

The epidemiological, anatomical and volumetric risk factors for dissection-related events were analyzed.

## 4. Statistical Analysis

All analyses were performed using the Statistical Package for Social Sciences software, version 20 (SPSS, IBM Corporation, New York, NY, USA). The medians and interquartile ranges (Q1–Q3) are used to describe the continuous variables; the categorical variables are presented as numbers and frequencies. The risk factors for an aneurysmal evolution and the distal reinterventions were assessed with a univariate analysis. The normality of the continuous variables was assessed by the Kolmogorov–Smirnov test. In cases of an abnormal distribution, Mann–Whitney tests were performed. In cases of a normal distribution, T-tests were performed. The categorical variables were compared by the χ^2^ or Fisher’s exact tests.

To identify the independent predictors for long-term dissection-related events, a binary logistic regression was conducted (descending method). The variables that were included in the model were selected based on the *p*-value obtained in the univariate analysis (*p* < 0.1) and were limited to four given the number of reported events. 

All statistical tests were 2-tailed, and *p*-values < 0.05 were considered statistically significant.

## 5. Results

### 5.1. Study Population

Between January 2010 and December 2016, 309 patients were treated in our center for a TAAD. Among them, the rate of stroke was 11.6% (36/309), the rate of a tamponade at admission time was 14.2% (44/309) and the in-hospital mortality was 17.7% (55/309).

Thirty-two patients did not have a RAD, and 48 patients were lost to follow-up.

Among the 174 survivors with a RAD, 38 patients died during follow-up (<3 years of follow-up) from non-aortic causes and without reoperation in the distal aorta and were excluded, 81 patients were excluded due to the absence of at least the 3 years follow-up or due to the absence of at least one of the three CT scan follow-ups during the first year.

Finally, 54 patients were included. 

The demographic data of the patients are presented in Table 1.

Following a median follow-up of 69.0 months (32–144), the rate of dissection-related events of the descending aorta was 62.9% (34/54). Among them, the median aortic diameter of the descending thoracic aorta was 57.0 mm (46–100). There were 12 aortic reinterventions (22.2%) for distal enlargement of the descending thoracic aorta and 3 postoperative deaths (2 perioperative deaths: 1 patient treated emergently for aortic rupture, 1 cardiac arrest and 1 late death secondary to an endoprosthesis infection 10 years later).

### 5.2. Measurement Validation

#### 5.2.1. Diameter

The intra- and interobserver intraclass correlation coefficients (CC) for the aortic diameter measurements (total aortic diameter) were greater than 0.96 and 0.92, respectively. The average difference was 0.5 mm between the readers across the measurements. No difference in the classification of the patients was found.

#### 5.2.2. Volume

The interobserver intraclass CC validated the TAV software in the calculation of the volumes of the TL (CC = 0.92) and the FL (CC = 0.96) (Supplemental Scheme S1).

The interobserver intraclass CC was greater than 0.94.

### 5.3. Anatomical Risk Factors for the Dissection-Related Events

#### 5.3.1. Univariate Analysis

##### Aortic Diameter Analysis

The total aortic diameters within the descending thoracic aorta were significantly greater in group 1 than in group 2 at T1, T2 and T3. This difference was linked to a greater diameter in the false lumen (Table 2).

An initial aortic diameter greater than 36 mm predicts the long-term risk of dissection aortic-related events with a specificity of 90% and a sensitivity of 75% (Supplemental Scheme S2).

##### Aortic Diameter Evolution Analysis

**Figure 3:**
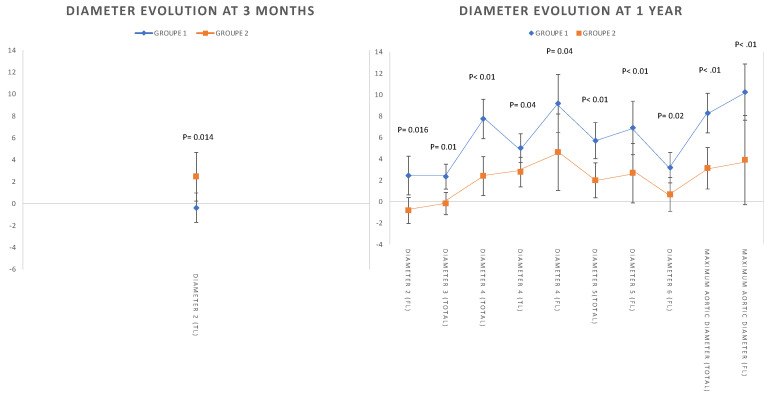
The aortic diameter evolution (true lumen (TL) and false lumen (FL)) at 3 months and 1 year: significant evolution in the aortic diameter within the different diameters.

The significant aortic diameter evolution at 3 months (T2) and 1 year (T3) is summarized in Figure 3.

The aortic diameter evolution at 3 months was not predictive of long-term dissection-related events. An aortic diameter evolution that is greater than 5 mm at 1 year predicts the long-term risk of dissection-related aortic events with a sensitivity of 70% and a specificity of 67% (Supplemental Scheme S2).

##### Entry Tear 

An entry tear > 10 mm (55.9% (19/34) vs. 25.0% (5/20); *p* = 0.027) was significantly associated with the risk of dissection related aortic events.

##### Volume Analysis

**Figure 4:**
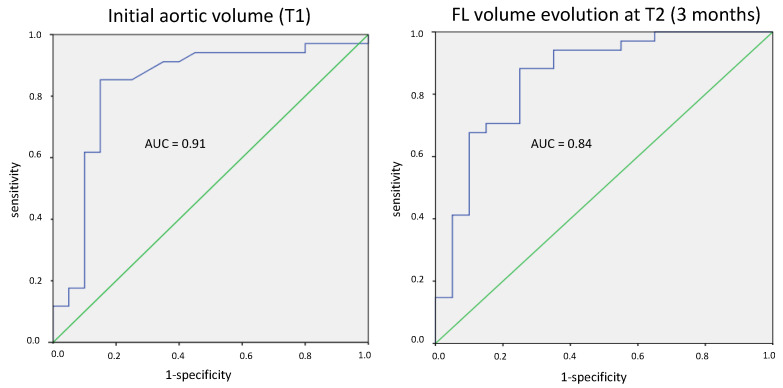
ROC curves showing: sensitivity and specificity of the initial false lumen (FL) volume at T1. Sensitivity and specificity of the FL volume evolution at 3 months.

The total aortic volume within the thoracic aorta was significantly greater in group 1 at T1, T2 and T3 and was directly related to the expansion of the FL (Figure 4); there was a significant difference in the volume of the FL at T1 (178 mL vs. 128 mL in groups 1 and 2, respectively, *p* < 0.01), T2 (235 mL vs. 129 mL in groups 1 and 2, respectively, *p* < 0.01) and T3 (255 mL vs. 131 mL in groups 1 and 2, respectively, *p* < 0.01). There was no significant difference in volume of the true lumen: 165 mL vs. 144 mL, 165 mL vs. 145 mL and 180 mL vs. 161 mL at T1, T2 and T3 in groups 1 and 2, respectively.

A volume of the FL that was > 140 mL on the initial CT scan predicted the long-term dissection-related events with a sensitivity of 85% and a specificity of 85% (Figure 4).

##### Volume Evolution Analysis

**Figure 5:**
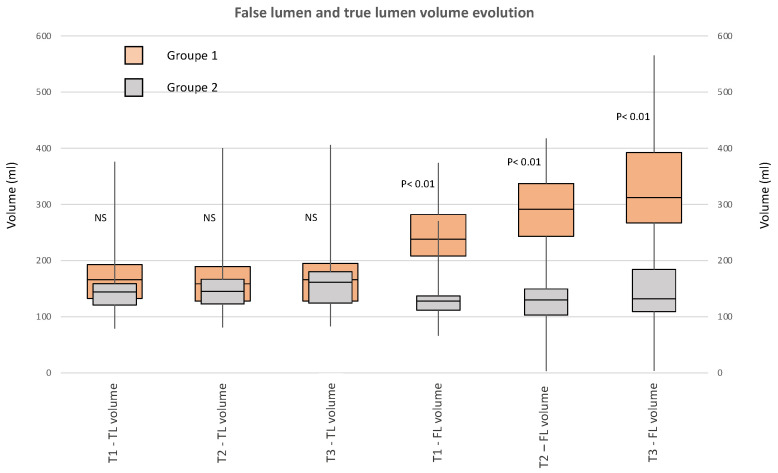
Volume of the true lumen (TL) and the false lumen (FL) evolution at 3 months and 1 year in groups 1 and 2. In group 1, the volume of the TL was stable over time, and the volume of the FL had increased significantly at 3 months and 1 year. In group 2, the volume of the TL had increased significantly at 3 months and 1 year, and the volume of the FL was stable over time.

The evolution of the aortic volume at 3 months (T2) and 1 year (T3) is summarized in Figure 5.

At 3 months and 1 year, the increase in the volume of the FL was significantly greater in group 1 than in group 2 (*p* < 0.01). There was no difference in the evolution of the TL.

A greater than 13% change in the volume of the FL at 3 months predicts the long-term dissection-related events with a sensitivity of 88% and a specificity of 75% (Figure 5).

#### 5.3.2. Multivariate Analysis

The initial aortic diameter was an independent predictive factor of long-term dissection-related events: HR = 1.49 CI_95_ = [1.05–2.10], *p* = 0.026. The initial aortic volume and the evolution of the FL volume at 3 months tends to be associated with the risk of long-term dissection-related events: HR = 1.03 CI_95_ = [0.99–1.07], *p* = 0.070; HR = 1.11 CI_95_ = [0.99–1.26], *p* = 0.078, respectively.

## 6. Comment

This is the first study to identify the aortic volume as a predictor of a long-term unfavorable evolution of a RAD after a TAAD. Recently, we published the results of a prospective follow-up study after a TAAD repair, and we showed that the RAD is associated with a high rate of aortic dissection-related events, which requires a close follow-up at an expert center [13].

Following a RAD, the risk factors for aortic growth in the descending thoracic aorta that have been identified in the literature, include young age, connective tissue diseases, bicuspid aortic valve, male sex, large initial aortic diameter, absence of resection of the primary entry tear during initial surgery or a new entry tear and the persistence of a patent FL [3,4,5,6,8,9,10,11,24,25]. The most relevant anatomical criteria found in the literature are the initial aortic diameter [3,4,5,6,8,9,10,11], the presence of a new entry tear on the distal anastomosis [6] or absence of a resection of the primary entry tear [3,4,5].

Our study confirms that the initial aortic diameter predicts a long-term unfavorable evolution, but the diameter alone, although confirmed in several studies, is not enough to screen patients in an optimal way. Furthermore, the contribution of the volumes in this context is major. Indeed, an aortic dissection involves the entire thoracic aorta, and the volume is therefore more suitable than the aortic diameter measurement at different levels. We evaluated the anatomy of the whole aorta with the TL and the FL analyzed separately, which confirmed that the unfavorable evolution occurs at the expense of the FL. More importantly, considering the anatomical evolution over time, the aortic volume determination allows us to predict the long-term fate of a RAD earlier than the aortic diameter. Indeed, we showed that the aortic FL volume evolution at just 3 months predicted a long-term unfavorable evolution with a sensitivity of 88% and specificity of 75%, whereas the evolution of the aortic diameter at 3 months was not predictive of long-term dissection-related events. Currently, many teams are working on the automation of the volume calculation with the use of artificial intelligence, which would make it possible to use the volumes in clinical practice.

We studied the anatomical criteria on the CT scans performed during the first year after a type A repair to propose an early endovascular treatment. Indeed, an endovascular repair of an acute or subacute dissection is associated with a rapid expansion of the TL and a collapse of the FL. In contrast, an endovascular treatment for a chronic dissection can induce a FL thrombosis in the treated segment without a change in the aortic diameter in most cases and with a patent FL within the thoraco-abdominal aorta [26]. The anatomical criteria identified in this study (initial aortic volume and diameter, aortic volume evolution at 3 months) coupled with the demographic factors that are associated with a poor prognosis of a RAD could be used in the future to screen patients at risk of an aneurysmal evolution, reinterventions and death, and propose a more aggressive treatment at an early stage to promote the aortic remodeling and improve long-term survival without reintervention.

Hemodynamic factors are also important to predict the long term evolution of a RAD [27,28] and improve the understanding of this complex pathology. In a recent study [29], we developed a numerical patient-specific modeling of a RAD and we performed a hemodynamic analysis on two patients. Although these comparisons could only be conducted on two patients and need to be confirmed by a larger number of cases, our findings point to these hemodynamic markers as possible candidates for an early evaluation of the pathology’s evolution towards an unfavorable scenario.

### Limits 

This was a retrospective monocentric study, which could limit the external validity of this study. The other limitations are the small study group and the number lost to follow-up, so the next step is to confirm our results in a prospective study.

The exclusion criteria could limit the interpretation of the results.

## 7. Conclusions

The first year after a TAAD is a critical period, but the increase in the volume of the FL at an early stage was significantly greater in patients with a long-term unfavorable evolution. The volume analysis in addition to the diameter analysis could be used in the future to screen patients at risk of an aneurysmal evolution, reinterventions and death, and propose a more aggressive treatment at an early stage to promote the aortic remodeling and improve long-term survival without reintervention.

## Figures and Tables

**Table 1:** Demographic data.

Variables	Total *n* = 54	Dissection Related Events *n* = 34	Non-Dissection Related Events *n* = 20	*p* Value
**Male sex, *n* (%)**	35 (64.8)	23 (67.6)	12 (60.0)	0.57
**Age, median (range)**	62.5 (37–82)	64.3 (37–82)	60.4 (40–75)	0.19
**Hypertension, *n* (%)**	34 (62.9)	22 (64.7)	12 (60.0)	0.72
**Dyslipidemia, *n* (%)**	6 (11.1)	3 (8.8)	3 (15.0)	0.66
**Smoking, *n* (%)**	18 (33.3)	9 (26.5)	9 (45.0)	0.16
**Diabetes, *n* (%)**	4 (7.4)	4 (11.8)	0 (0)	0.29
**COPD, *n* (%)**	2 (3.7)	1 (2.9)	1 (5.0)	>0.99
**CAD, *n* (%)**	2 (3.7)	1 (2.9)	1 (5.0)	>0.99
**Renal failure, *n* (%)**	1 (1.8)	0 (0.0)	1 (5.0)	0.37
**Marfan syndrome, *n* (%)**	1 (1.8)	1 (2.9)	0 (0)	>0.99
**Bicuspid aortic valve, *n* (%)**	1 (1.8)	0 (0)	1 (5.0)	0.37
**Follow-up (months), median (range)**	69.0 (32–144)	76.0 (32–131)	66.5 (34–144)	0.47
**Initial type A aortic repair, *n* (%)**				
Ascending aortic replacement	2 (3.7)	1 (2.9)	1 (5.0)	0.05
Hemiarch replacement	42 (77.8)	24 (70.6)	18 (90.0)	
Partial aortic arch replacement	6 (11.1)	5 (14.7)	1 (5.0)	
Total aortic arch replacement	4 (7.4)	4 (11.8)	0 (0.0)	

COPD: Chronic obstructive pulmonary disease, CAD: coronary artery disease, SD: standard deviation.

**Table 2:** Comparison of the measurement of the diameter at each level of the thoracic aorta between groups 1 and 2 at T1 (postoperative CT scan), T2 (3 months) and T3 (1 year).

		T1			T2			T3		
	Levels	Group 1	Group 2	*p*-Value	Group 1	Group 2	*p*-Value	Group 1	Group 2	*p*-Value
Total aortic diameter mm, med (range)	D 1	35.1 [32.4–40.9]	34.0 [29.0–38.7]	0.201	35.5 [32.4–40.2]	33.8 [30.8–37.8]	0.186	35.8 [31.9–41.9]	32.9 [30.0–39.9]	0.116
	D 2	43.1 [36.1–45.4]	37.3 [33.7–41.0]	0.033 *	41.7 [36.4–48.0]	37.9 [33.3–40.7]	0.023 *	43.4 [36.5–51.6]	37.4 [31.7–42.7]	0.018 *
	D 3	40.9 [35.7–44.9]	35.2 [34.1–37.5]	0.001 *	41.4 [36.3–46.3]	36.2 [32.5–37.0]	<0.001 *	44.6 [36.0–47.5]	35.9 [31.3–38.8]	<0.001 *
	D 4	39.5 [36.9–42.9]	34.1 [31.0–36.8]	<0.001 *	44.0 [40.1–49.3]	34.4 [32.5–38.1]	<0.001 *	48.4 [43.6–53.5]	37.5 [32.9–40.4]	<0.001 *
	D 5	35.8 [33.4–38.7]	31.8 [29.5–34.1]	<0.001 *	39.0 [35.2–43.3]	32.2 [29.2–34.3]	<0.001 *	42.2 [36.8–46.2]	34.3 [30.2–37.0]	<0.001 *
	D 6	31.8 [30.2–34.6]	29.3 [27.7–32.6]	0.009 *	33.0 [31.7–36.5]	30.0 [28.7–33.1]	0.001 *	35.4 [33.2–37.7]	30.7 [29.1–34.0]	<0.001 *
True lumen mm, med (range)	D 1	34.2 [30.9–37.5]	31.5 [28.6–35.7]	0.380	35.7 [31.6–38.6]	32.5 [28.6–37.7]	0.158	36.0 [33.7–41.0]	35.2 [32.2–39.7]	0.814
	D 2	36.4 [35.2–39.2]	31.5 [27.2–34.8]	0.001 *	35.9 [34.4–40.0]	34.5 [28.4–36.1]	0.034 *	37.0 [34.1–40.7]	33.8 [30.3–36.5]	0.020 *
	D 3	34.6 [30.7–38.1]	29.2 [27.4–32.0]	<0.001 *	36.8 [32.0–40.2]	31.2 [29.7–32.8]	0.001 *	37.2 [32.8–40.8]	30.4 [28.9–32.8]	<0.001 *
	D 4	28.4 [25.5–32.0]	27.2 [23.8–29.2]	0.209	32.0 [27.7–36.3]	28.1 [26.5–31.3]	0.025 *	33.3 [29.3–37.2]	29.1 [26.6–32.1]	0.003 *
	D 5	27.8 [23.1–30.5]	24.8 [23.5–28.6]	0.352	28.4 [25.2–30.3]	26.7 [24.2–31.4]	0.599	29.5 [27.0–32.1]	27.5 [26.4–29.1]	0.051
	D 6	25.7 [23.4–28.0]	24.2 [20.4–27.9]	0.244	27.0 [24.3–29.3]	26.2 [21.7–29.2]	0.565	27.3 [25.6–30.8]	27.2 [23.9–28.5]	0.629
False lumen mm, med (range)	D 1	32.8 [17.2–40.5]	35.7 [32.2–37.8]	0.526	39.7 [26.9–48.5]	33.6 [20.2–40.8]	0.386	40.5 [24.1–52.7]	36.3 [24.7–41.0]	0.409
	D 2	37.1 [32.9–40.7]	33.2 [26.6–36.6]	0.043 *	37.4 [30.7–43.4]	34.5 [29.6–36.1]	0.086	38.2 [32.9–43.4]	33.8 [29.4–37.3]	0.053
	D 3	36.6 [31.8–40.2]	29.6 [26.6–33.4]	0.001 *	36.5 [31.3–41.1]	31.0 [26.7–32.5]	0.002 *	37.2 [31.6–41.4]	32.1 [27.7–33.4]	0.002 *
	D 4	39.1 [35.4–41.8]	34.1 [30.9–35.7]	0.001 *	42.4 [38.7–48.5]	34.6 [32.1–38.9]	<0.001 *	45.3 [41.6–51.8]	37.3 [32.9–38.4]	<0.001 *
	D 5	35.0 [33.1–38.7]	31.7 [29.2–33.3]	<0.001 *	38.3 [34.3–40.9]	32.3 [29.7–33.9]	<0.001 *	39.9 [35.5–45.8]	33.4 [30.8–35.5]	<0.001 *
	D 6	31.3 [29.1–33.7]	28.5 [26.3–32.1]	0.025 *	32.7 [31.1–35.7]	29.3 [27.3–31.5]	<0.001 *	33.9 [32.1–36.8]	28.7 [27.3–32.5]	0.001 *

D: diameter * Statistically significant Med: median.

## Data Availability

The data underlying this article will be shared upon reasonable request to the corresponding author.

## Supplementary Materials

The following supporting information can be downloaded at: https://www.mdpi.com/article/10.3390/jcdd9100349/s1, Scheme S1: Validation of the custom application of the TAV results. Comparison of the results obtained using a reference method by another observer [23,24,25]. Scheme S2: ROC curve showing the sensitivity and specificity of the total aortic diameter measurement at T1. ROC curve showing the sensitivity and specificity of the aortic diameter evolution at 3 months (blue) and 1 year (green).

## Abbreviations

RAD	residual aortic dissection
TAAD	type A aortic dissection
CL	centerline
CT	computed tomography
FL	false lumen
TL	true lumen
EV	elementary volume
CC	coefficient correlation
HR	hazard ratio
