# Supplementary material for: Volume Analysis to Predict the Long-Term Evolution of Residual Aortic Dissection after Type A Repair
Source: J Cardiovasc Dev Dis. 2022 Oct 12;9(10):349. doi: 10.3390/jcdd9100349 (PMC9604488; doi:10.3390/jcdd9100349)
Supplement: Supplementary file 1 [file jcdd-09-00349-s001.zip › jcdd-1903373-supplementary.pdf]

## Supplemental figure legends

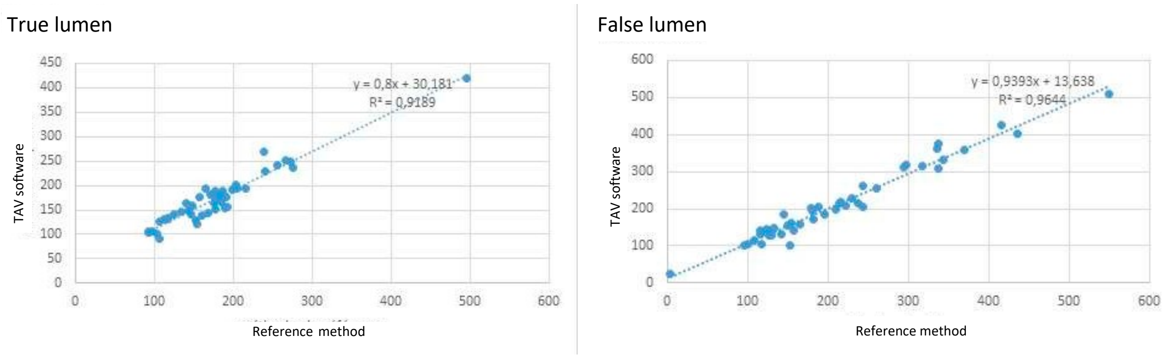


**Scheme S1.** Validation of the custom application TAV results.

Comparison of the results obtained using a reference method by another observer [23–25].

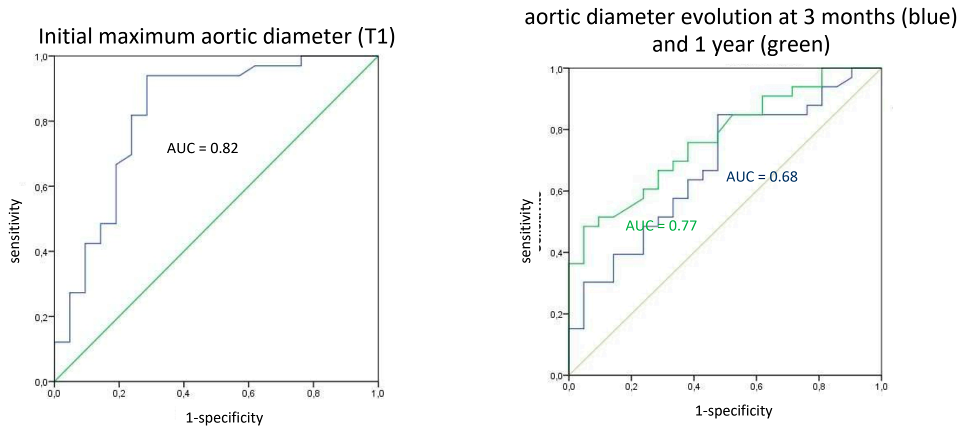


**Scheme S2.** ROC curve showing the sensitivity and specificity of the total aortic diameter measurement at T1.

ROC curve showing the sensitivity and specificity of the aortic diameter evolution at 3 months (blue) and 1 year (green).

## References


23. England, A.; Fisher, R.; McWilliams, R.; Torella, F. Estimating the error of CT-based measurements of aortic lumen volume used in endovascular planning. Radiography 2017, 23, 287–291.

24. Concistrè, G.; Casali, G.; Santaniello, E.; Montalto, A.; Fiorani, B.; Dell'Aquila, A.; Musumeci, F. Reoperation after surgical correction of acute type A aortic dissection: Risk factor analysis. Ann. Thorac. Surg. 2012, 93, 450–455.

25. Kimura, N.; Tanaka, M.; Kawahito, K.; Yamaguchi, A.; Ino, T.; Adachi, H. Influence of patent false lumen on long-term outcome after surgery for acute type A aortic dissection. J. Thorac. Cardiovasc. Surg. 2008, 136, 1160–1166, 1166 e1–e3.
